# The Prevalence of Attention-Deficit Hyperactivity Disorder in Functional Neurological Disorder: An Integrative Literature Review

**DOI:** 10.1192/bjo.2024.252

**Published:** 2024-08-01

**Authors:** Catriona Staunton, Roopa Rudrappa, Mohanbabu Rathnaiah

**Affiliations:** 1 University of Nottingham, Nottingham, United Kingdom; 2 Derbyshire Healthcare NHS Foundation Trust, Derby, United Kingdom

## Abstract

**Aims:**

Functional Neurological Disorders (FNDs) affect motor or sensory functions without a detectable underlying disease. FNDs encompass a range of presentations including non-epileptic seizures, cognitive changes, weakness, and sensory symptoms. The prevalence of FND as a diagnosis is increasing rapidly. Following our clinical observations of a high prevalence of Attention-Deficit Hyperactivity Disorder (ADHD) in people referred with a previous diagnosis of FND to our tertiary Neuropsychiatry pilot service in Derbyshire, we conducted an integrative literature review with the aim to investigate the prevalence of ADHD in people diagnosed with FND.

**Methods:**

We conducted an integrative literature review using a systematic approach. A literature search was performed on two databases, PubMed and ScienceDirect. The keywords ‘Functional Neurological Disorder’, ‘Attention-Deficit Hyperactivity Disorder’, ‘Non-Epileptic Seizures’, ‘Functional’ were used. Databases were searched for initial search on 31 November 2023 and the search was repeated on 31 January 2024. Only articles in English language were included. Studies were eligible if reporting the prevalence of ADHD in FND populations. Studies involving adults and children were included. A further search was conducted on reference lists from the selected articles.

**Results:**

Database searches on PubMed and ScienceDirect had 298 and 11,837 results, respectively. Only seven studies were identified that explored the prevalence of ADHD in individuals diagnosed with a FND and were included. In the adult population an association between a FND diagnosis, and ADHD traits identified on screening, or a final ADHD diagnosis was identified. The findings also demonstrate an increased incidence of comorbid ADHD and FND with the presence of another co-existing neurodevelopmental disorder such as Autism Spectrum Disorder. Furthermore, results indicated that the prevalence of an ADHD diagnosis in children with a FND was higher compared with adults. The literature suggests that, in both adults and children with FND-related functional seizures there is an increased prevalence of comorbid ADHD.

**Conclusion:**

In conclusion, the findings from this review demonstrate a lack of evidence looking into the prevalence of Attention-Deficit Hyperactivity Disorder in complex presentations being labelled as Functional Neurological Disorder. However, the existing literature indicates there is an association between FND and ADHD. These findings highlight the importance of considering potential ADHD comorbidity in the assessment and management of FND, potentially informing targeted treatment approaches for affected individuals. Further research could explore the efficacy of ADHD medication and similar dopamine modulating molecules in treating sub-cohort of people with FND.

